# How Chinese Consumers’ Intentions for Purchasing Eco-Labeled Products Are Influenced by Psychological Factors

**DOI:** 10.3390/ijerph17010265

**Published:** 2019-12-30

**Authors:** Jie Jin, Qiuhong Zhao, Ernesto DR Santibanez-Gonzalez

**Affiliations:** 1 School of Economics and Management, Taiyuan University of Technology, Taiyuan 030000, China; jinjie18701241953@foxmail.com; 2 School of Economics and Management, Beihang University, Beijing 100191, China; 3 Beijing Key Laboratory of Emergency Support Simulation Technologies for City Operations, Beijing 100091, China; 4 Beijing International Science and Technology Cooperation Base for City Safety Operation and Emergency Support, Beijing 100091, China; 5 Department of Industrial Engineering, Faculty of Engineering, University of Talca, Curico 3340000, Chile; santibanez.ernesto@gmail.com

**Keywords:** theory of planned behavior, eco-labeling, environmental awareness, health consciousness, structural equation modelling

## Abstract

This paper studies how consumers’ psychological factors influence their intentional purchasing behavior towards eco-labeled products and investigates why consumers choose eco-labeled products. Based on the Theory of Planned Behavior, we develop an extended model including six constructs. Among these constructs, consumers’ intentional purchasing behavior, attitude towards the behavior, subjective norm, and perceived behavioral control are applied from the original theoretical framework. Health consciousness and environmental awareness are integrated additionally to reflect consumers’ concerns about the natural environment and their health. Next, we conduct and analyze a survey-based empirical study with 336 samples using the Structural Equation Modeling. Our findings show that consumers’ attitude towards the behavior is positively influenced by environmental awareness, but the effects of it on intentional purchasing behavior are insignificant. Also, subjective norm has a positive influence on intentional purchasing behavior, but its effects on attitude towards the behavior are not significant. Moreover, the results also show that the total effects of health consciousness on intentional purchasing behavior are significantly higher than the effects of environmental awareness. Our results can provide a reference for business managers to attract consumers through eco-labeling as well as government policymaking.

## 1. Introduction

Recently, several studies and researches have suggested that consumers prefer products produced under environmentally friendly practices [1,2,3]. Compared with conventional products, these “green” or “clear” products are required to use greener materials and ways of production, and they would have less harmful impacts on the natural environment [4,5,6]. To cater for the green tastes and needs of consumers, more and more producers choose to invest in more sustainable practices [7,8].

The eco-label is used to validate that a product is up to the corresponding environmental standards formulated by government agencies and other public service organizations [9]. For example, Type I labels (ISO 14024) require that the whole process of a product including production, transportation, marketing, and recycling is in keeping with environmental standards and harmless both to the natural environment and to public health [10,11]. Eco-labeling schemes are helpful to eliminate the information asymmetry [12,13] between producers and consumers. Through eco-labels, consumers could be informed about the environmental effects of the products and encouraged to reduce environmental damages by changing their purchasing choices. Eco-labeling schemes are beneficial for reducing negative environmental impacts and promoting green consumptions [14].

Though it has been a consensus that eco-labeling schemes are helpful for making a change towards more eco-friendly consumption patterns [15,16], eco-labels could be effective only if they have actual effects on consumer’s decision-making process [17]. With regard to researches about influential factors of consumers’ purchasing intentions for eco-labeled products in developed countries and some developing countries, many researchers have proved that environmental awareness plays an important role when consumers purchase eco-labeled products [8,18,19,20]. However, the situation is somewhat different in some developing countries. Taking China as an example, due to the extensive food and commodity safety accidents that were exposed by the media in recent years, consumers are increasingly concerned about the products’ qualities when they choose them [21,22,23]. Health consciousness has been an important factor motivating consumers to choose eco-labeled products, even if they have to pay a premium [24,25]. Hence, it is necessary to investigate the motivations for Chinese consumers purchasing eco-labeled products and the corresponding influences on consumers’ preferences.

To this purpose, based on the Theory of Planed Behavior (TPB) [26,27], a conceptual model including six constructs is built in this paper. Among these constructs, subjective norm (SN), perceived behavioral control (PBC), attitude towards the behavior (ATB), and intentional purchasing behavior (IPB) come from TPB. To make up the deficiency that TPB ignores one’s needs prior to engaging in a certain action which can influence behaviors regardless of expressed attitudes, health consciousness (HC) [28] and environmental awareness (EA) [29] are integrated to the model to reflect consumers’ concerns about the natural environment and their health.

As a private motive, health consciousness captures consumers’ concern for their own health and safety. In general, a higher standard would be required for eco-labeled products, and the production processes of them would receive more strict supervision from the society and media. Hence, some consumers tend to think that eco-labeled products have a higher quality compared with ordinary products. And these consumers purchase eco-labeled products with a consideration of their personal health and food safety [30] In contrast to health consciousness, consumers’ environmental awareness reflects the degree of their attentions paid to common natural environment. According to the Externality Theory, environmental awareness is viewed as an altruistic motive for the positive externalities of environmental behavior [31]. For environmentally conscious consumers, they tend to choose product with little damaging on the environment, even though they need to pay a high price [32].

In terms of the conceptual model, we aim at analyzing psychological factors influencing consumers’ intentions for eco-labeled products in China and investigate to what extent consumers’ intentional purchasing behaviors are influenced by their private (benefits the individual) and altruistic (benefits the public environment) motives. Specifically, we address the following research questions:

**Question** **1.** *How social norm (SN), environmental awareness (EA), health consciousness (HC), perceived behavioral control (PBC), and attitude towards the behavior (ATB) influence consumer’ intentional purchasing behavior (IPB)?*

**Question** **2.** *For their own health and safety or for the natural environment, which play a more important role when consumers choose eco-labeled products? Namely, does there exist a statistically significant difference between the total effects of HC and EA on IPB?*

The remainder of this paper is organized as follows. In Section 2, we present the research background. In Section 3, we introduce the important concepts and develop the extended TPB framework. In Section 4, we illustrate the methodology issues including measure development, data collection, reliability, and validity tests. Section 5 reports the results of structural model analysis. In Section 6, we conclude with a discussion of our findings, policy and theoretical implications, limitations of the research, and avenues for future researches.

## 2. Background

### 2.1. Theoretical Background

Eco-labeling is viewed as a useful tool to promote the development of green consumption. More eco-friendly consumption patterns could be built through eco-labeling schemes [15,33]. For the negative internality of environmental actions, an enterprise would not take costly environmental measures on its own [34,35]. As an important channel between manufacturers and consumers for the green information transmission, consumers could be informed about the environmental efforts of the producers through eco-labeling [14,36].

Although the development of eco-labeling schemes is advocated by many governments around the world, the purchases of eco-labeled products in the market are not so good [37,38]. As the most important participants of green consumption, consumers play a crucial role in pollution and emission reduction [39]. All incentive policies of the government and environmental efforts of enterprises are effective only when they factually promote consumers’ green purchasing behaviors [40]. Hence, it is necessary to analyze the influencing factors of consumers’ purchasing behaviors towards eco-labeling products and provide promotion measures.

From the point of green consumption, it is a kind of environmental purchasing behavior for consumers buying eco-labeled products [15]. According to corresponding researches, there are many factors that can have an influence on consumers’ green purchasing behaviors. For consumers, these factors can be divided into internal factors and external factors, of which external factors include influences from the government, the enterprise, and the news media [38,41], and the internal factors include the demographics factors and psychological factors [17,42].

In this paper, we focus on the influences of psychological factors. Because, on the one hand, psychological factors like attitude towards green consumption, environmental awareness, social norm and so forth are the internalization of external factors [31,43]. On the other hand, although some researchers have claimed that demographics factors like income, gender, and education can have an influence on consumers’ green purchasing behaviors [44], some other researchers find that influences of these factors are not always consistent in different researches, and are even conflicted in some situations. Hence, some researchers think that demographics factors are not determining factors in consumers’ green purchasing behaviors [42].

In reality, except for the considerations for environmental issues, consumers would also purchase eco-labeled products for other issues. In general, the production processes of eco-labeled products would implement a higher standard and receive more strict supervision from the society and media at the same time. Hence, consumers tend to think that eco-labeled products have a higher quality compared with parallel products and purchase eco-labeled products with a consideration of their personal health and food safety [24,45].

Moreover, we should notice that the existing eco-labeling schemes in different countries are very different, and different certification systems and standards would result in different interaction relationships among stakeholders [46]. Regarding this point, we focus on Chinese Environmental Labeling (CEL), which is an eco-labeling scheme sponsored by the Chinese government. It is also one of the most influential and authoritative eco-labeling schemes in China [38]. To help readers understand the research background better, we give a brief introduction to CEL in Section 2.2.

### 2.2. A Description of the Chinese Environmental Labeling Scheme

The Chinese Environmental Labeling (CEL) scheme was launched by the Ministry of Environmental Protection of the People’s Republic of China (MEP) in 1993. At present, about 4000 enterprises and 200,000 products have been verified. From August 2001, CEL started to follow the regulations of ISO 14024. Compared with other eco-labeling schemes in China, CEL requires that labeled products are not only qualified but also have fewer environmental impacts than parallel products. It is a full life-cycle activity scheme considering from product design, production, packaging, and transportation to consumption and recirculation [38].

The general certification process of CEL is as follows: A prospective enterprise submits a request as well as the required testimonial materials to the China Environmental United Certification Center (CEC). If qualified, the enterprise needs to pay the certification fees and signs a contract with the CEC. Then, a check panel would be allocated by the CEC to the enterprise’s actual production field. The panel conducts on-site investigations of the enterprise and sends selected samples to a CEC-affiliated testing organization for further testing. Based on the materials submitted by the enterprise, results of the field investigation, and sample test results from the testing organization, the check panel makes a report and submits it to the technical committee of the CEC. The technical committee summarizes the results of the investigation and decides on whether the eco-label is granted.

**Figure 1:**
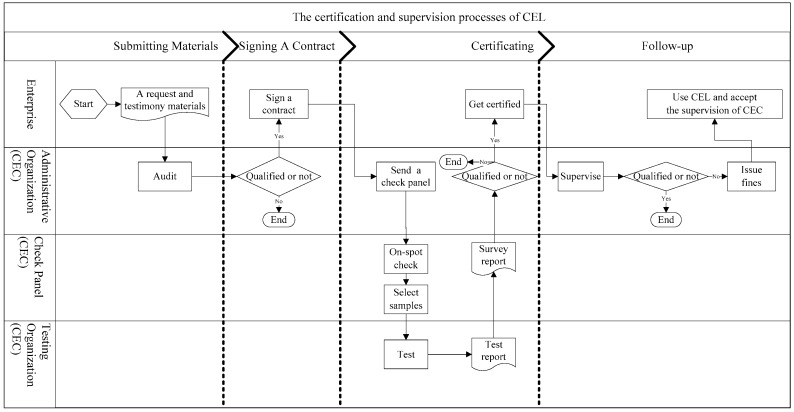
The certification and supervision processes of CEL (Chinese Environmental Labeling).

If approved, the enterprise would be authorized to use CEL for three years. The enterprise can print the label on the packaging of its products. At the same time, labeled products have priority for government procurement (in 2006, the Ministry of Finance of the People’ s Republic of China (MOF) issued rules requiring government agencies to give priority to CEL products). In general, the CEC would inspect the enterprise annually, and the local Administrations for Industry and Commerce (AIC) also has supervision duties. The supervision results of the AIC would be reported to the CEC. If an enterprise was found not to implement the standards, the CEC would issue fines to it. After three years, the enterprise needs to resubmit materials and accepts recertification [20]. The specific processes are presented in Figure 1 [38].

From Figure 1, we can see that the certification and supervision process design of CEL seems to be rigorous. Compared with ordinary products, a higher standard is required for eco-labeled products and more supervisions from government agencies would be applied to the production processes. Hence, an inherent idea would be imposed to consumers and makes them believe that eco-labeled products have a higher quality and safety status.

## 3. The Conceptual Framework and Hypotheses

### 3.1. The Conceptual Framework

Based on the TPB and some empirical studies in the green consumption context, an extended model which includes six constructs is developed. The six constructs are subjective norm (SN), environmental awareness (EA), health consciousness (HC), perceived behavioral control (PBC), intentional purchasing behavior (IPB), and attitude towards the behavior (ATB). Among them, SN, PBC, IPB, and ATB are applied from TPB [27]. EA and HC are newly added and are two important psychological factors when consumers make purchasing choices [24,30,45,47].

**Figure 2:**
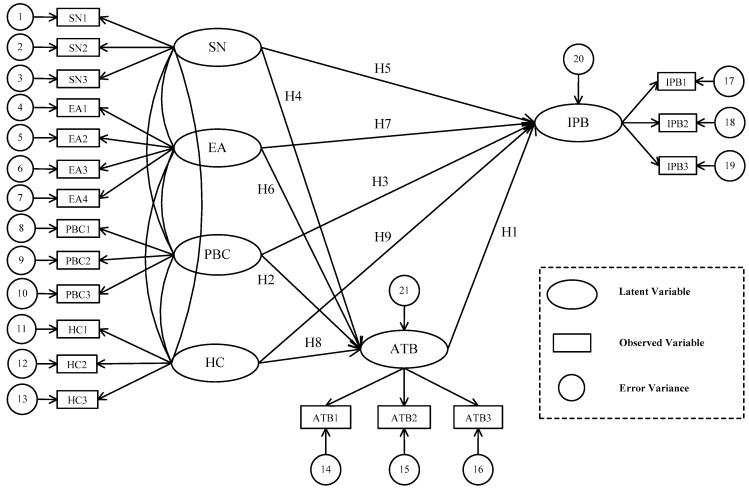
Conceptual Framework. SN: subjective norm. EA: environmental awareness. PBC: perceived behavioral control. HC: health consciousness. IPB: intentional purchasing behavior. ATB: attitude towards the behavior. H: hypothesis.

As shown in Figure 2, nine single-headed arrows represent the hypothetical relationships from the measured latent variables at the tail of the arrow to the measured latent variables at the point. Nine hypotheses are summarized as below: (H1) Consumers’ attitude towards purchasing eco-labeled products has a positive effect on their intentional purchasing behaviors; (H2) Perceived behavioral control has a positive influence on consumers’ attitude towards purchasing eco-labeled products; (H3) Perceived behavioral control has a positive influence on consumers’ intentional purchasing behaviors; (H4) Subjective norm has a positive influence on consumers’ attitude toward purchasing eco-labeled products; (H5) Subjective norm has a positive influence on consumers’ intentional purchasing behaviors; (H6) Environmental awareness has a positive influence on consumers’ attitude toward purchasing eco-labeled products; (H7) Health consciousness has a positive influence on consumers’ intentional purchasing behaviors; (H8) Health consciousness has a positive influence on consumers’ attitude toward purchasing the eco-labeled products; (H9) Health consciousness has a positive influence on consumers’ intentional purchasing behaviors. 

A total of 19 observed variables (measurement items) (SN1~SN3, EA1~EA4, PBC1~PBC3, HC1~HC3, ATB1~ATB3, IPB1~IPB3) for the six latent variables are developed. As depicted in Figure 2, ovals represent latent variables, rectangles represent observed variables, and circles represent error variables. The theoretical bases and empirical verifications of these hypotheses are presented in Section 3.2. The 19 measurement items were adopted from the previous relevant researches, the detailed descriptions of them are present in Section 4.1.

### 3.2. Hypotheses

#### 3.2.1. The Theory of Planned Behavior

Purchasing intention is a consciously decided plan to buy a product or service [39]. Purchasing intention is commonly used to predict purchasing behavior and considered as the most suitable tool for predicting the behaviors of consumers under many situations, including the green consumption. In consideration of the difficulty to assess the actual purchasing behaviors, purchasing intention is usually taken as a proxy of actual purchasing behavior [48].

The Theory of Planned Behavior (TPB) was proposed by Ajzen in 1985, and it is considered as a theory that links one’s belief and behavior. TPB extends from the Theory of Reasoned Action, with adding a new component: “perceived behavioral control”. By this, TPB can cover nonvolitional behaviors for better predicting behavioral intentions and actual behaviors [27].

According to TPB, attitude towards the behavior, subjective norms, and perceived behavioral control all together form behavioral intentions [32]. Among them, attitude is a psychological tendency which relates to the evaluations of consumers towards having the behavior, it is a stable, evaluative response to an entity [49]. Based on the researches of consumer behavior, attitude plays a strong role in influencing the behavior [39]. The more positive consumers feel about purchasing a product, the higher probability they would like to purchase it. The relationship between consumers’ attitude and behavioral intention has been confirmed in many researches [21,32]. The existing evidences led us to develop the following hypothesis:

**Hypothesis** **1** **(H1).** *Consumers’ attitude toward purchasing eco-labeled products has a positive effect on their intentional purchasing behavior.*

Perceived behavioral control reflects the ease or difficulty perceived by an individual when he/she performs a particular behavior [27]. Perceived behavioral control is determined by the total set of accessible control beliefs. From the perspective of a consumer who is considering choosing eco-labeled products, the total set of accessible control beliefs includes an affordable price, the availability to buy, and the accessibility about certain information [50,51]. If consumers perceive more availability, there would be less deterrents for them choosing eco-labeled products. Hence, we propose the following hypotheses.

**Hypothesis** **2** **(H2).** *Perceived behavioral control has a positive influence on consumers’ attitude towards purchasing eco-labeled products.*

**Hypothesis** **3** **(H3).** *Perceived behavioral control has a positive influence on intentional purchasing behavior.*

#### 3.2.2. Subjective Norm

Subjective norm refers to an individual’s perception about the particular behavior, which is influenced by the judgment of significant others (e.g., parents, spouse, friends, teachers) [52]. Given that consumers’ behavior might be in and dependent on a particular social network and organization, the attitudes and intentional behaviors of them are influenced by their friends, family, and the society [53].

The concept of subjective norm is conceptualized within collectivistic culture-related space and introduced to reflect the impacts of social influence. Subjective norm depicts the decision-making processes of consumers, that they would make an evaluation of whether their behaviors are expected and recommended by significant others. Thus, the following hypotheses are proposed:

**Hypothesis** **4** **(H4).** *Subjective norm has a positive influence on consumers’ attitude towards purchasing eco-labeled products.*

**Hypothesis** **5** **(H5).** *Subjective norm has a positive influence on intentional purchasing behavior.*

#### 3.2.3. Environmental Awareness

Consumerism is the consumption idea and consumption culture in the age of industry civilization. Consumerism is characterized by natural resources exhausting and environmental pollution and regarded as the main cause of the destruction of the ecosystem and environmental pollution [54]. Since 1990s, different studies have concluded that citizens rated the environment as an immediate and urgent problem to be addressed [55,56]. Nowadays, because of the growing awareness of the problematic relationship between modern industrialized society and the natural environment, consumers become more concerned about the influences of their daily activities on the environment [57,58].

From the perspective of the Value-Belief-Norm (VBN) theory in social psychology, the general environmental values, beliefs, and norms have a positive impact on pro-environmental behaviors [59]. VBN helps to explain the supportive behaviors of the public to social environmental movement. Researches from the Social Identify Theory (SIT) also provide evidences to the relationship between consumers’ environmental awareness and their intentions and behavior, where environmental awareness is viewed as a kind of positive social identification and people engage in environmental behaviors to gain acceptances [60].

The positive relationships among environmental awareness and pro-environmental intentions and behaviors have been proved by previous studies among different cultures and samples all over the world. Consumers with favorable environmental awareness are more likely to make environmentally conscious consumption decisions [4,32,51]. Based on the above discussions, we deduce the following hypotheses.

**Hypothesis** **6** **(H6).** *Environmental awareness has a positive influence on consumers’ attitude towards purchasing eco-labeled products.*

**Hypothesis** **7** **(H7).** *Environmental awareness has a positive influence on intentional purchasing behavior.*

#### 3.2.4. Health Consciousness

According to the Motivational Theory, the purchasing motivations of consumers can be divided into psychological motives and physiological motives. Among them, the physiological motive refers to a consumer’s pursuit of functional characteristics of intentional products, and a consumer’s psychological buying motive is the response to his/her specific psychological needs [61]. Health consciousness captures consumers’ concerns for their self-health status. To satisfy the needs to sustain, protect, extend, and develop the life, physiological motive constitutes the major driving force for consumers choosing eco-labeled products [62,63].

According to Maslow’s hierarchical theory of needs [64], product functionality should meet the physical and security needs of consumers. In China, as a lot of scandals about product safety and commodity quality have been exposed in recent years, the public are increasingly concerned about the product safety and quality [21,23]. In many consumers’ perceptions, eco-labeled products are required to implement a higher standard than the general products, therefore, the security and reliability of them are more likely to be trusted [38]. Considerations for health and safety have been an important influential factor in prompting green consumption. Thus, we predict the following hypotheses:

**Hypothesis** **8** **(H8).** *Health consciousness has a positive influence on consumers’ attitude toward purchasing eco-labeled products.*

**Hypothesis** **9** **(H9).** *Health consciousness has a positive influence on intentional purchasing behavior.*

## 4. Methodology

### 4.1. Data Collection

The data collection was conducted through a web-based questionnaire survey from October to December 2018. The potential respondents were approached via social media group channels like QQ (QQ is one of the most popular social softwares in China). By 2018, QQ have more users than other social software in China [65] and Wechat. Firstly, the research assistant asked the respondent if he/she had bought eco-labeled and explained the details about the aim of this survey. If the respondent had such experience and was willing to participate, an Invitation letter with a questionnaire link would be send to him/her.

In order that respondents can know the research background better, the questionnaire we used is constituted by three parts. In the part I of the questionnaire, we briefly introduce the knowledge about the eco-labeling scheme and eco-labeled products. Part II surveys the respondents’ demographic information. Part III collects data about consumers’ subjective norm, perceived behavioral control, attitude towards the behavior, environmental awareness, health consciousness, and intentional purchasing behavior.

The measured items for each construct are presented in Table 1. All constructs were measured with multiple-item scales. A total of 19 items for the six constructs used in this study were adopted from the related literature and modified to suit the study. Items to measure the six constructs are rated by a 7-point Likert scale (strongly disagree = 1; medium disagree = 2; disagree = 3; neutral/undecided = 4; agree = 5; medium agree= 6; strongly agree = 7).

A total of 500 consumers who had bought eco-labeled products were contacted, with 341 responses and 336 valid completed surveys. The valid response rate is 67.2%, which is above the 60 percent response rate recommended by Babbie [70]. According to Nunnally [71] along with Tabachnick and Fidell [72], the sample size of study (336) is enough to test measurement scales. We tested the normality of the distribution for variables including SN1~SN3, EA1~EA4, PBC1~PBC3, HC1~HC3, ATB1~ATB3, IPB1~IPB3. The results showed that the values of skewness and kurtosis of the scores are between −1.5 and 1.5, which is acceptable to prove the scores are univariate normal distributions [73].

The demographic information of the sample is presented in Table 2. Of those, responses are balanced among the sexes (male: 52.1%, female: 47.9%); the respondents’ ages range from 18 to 76. Of respondents, 36.3% are between 25–34 years of age, the highest proportion of all age groups. The next is 35–44, making up 25.9% of all respondents. Of the respondents, 86.3% hold the bachelor’s degree, and 51.8% of respondents’ income per month is between 10 K and 15 K.

### 4.2. Measurement Model Validation

In this paper, we apply the structural equation modeling (SEM) to analyze the relationships among six constructs (EA, HC, ATB, PBC, IPB, and SN). SEM is a kind of statistical approach which can analyze the relationships among variables through their covariance matrix [74]. In the field of sociology, psychology, and other social sciences, some concepts like intelligence, learning motivation, and social status cannot be measured directly, they are called latent variables. Compared with traditional statistical approaches, SEM can effectively handle latent variables and their indicators (observed variables) [75]. In recent years, SEM has been widely used in the sciences, business, education, and other fields [76].

SEM involves two main components: the measurement model showing the relationships among latent variables and their indicators, and the structural model showing potential causal dependencies among latent variables. The measurement model is evaluated through a confirmatory factor analysis (CFA). If the analysis results of CFA show that observed variables are suitable for measuring latent variables, then the relationships among latent variables could be analyzed through structural model [77].

As depicted in Figure 2, a confirmatory factor analysis model with six latent constructs variables and 19 observed variables measures was built. In this paper, we use composite reliability (CR) and average variance extracted (AVE) to test the reliability and convergent validity of the measurement model. As shown in Table 3, all standardized factor loadings are greater than 0.70, meeting the acceptable value of 0.50. The composite reliability (CR) scores range from 0.78 to 0.94, exceeding the threshold value of 0.70, suggesting good convergent validity. The average variance extracted (AVE) values range from 0.54 to 0.84, which are above the acceptability value of 0.50, indicating convergent validity at the construct level [78].

Satisfactory discriminant validity of the measure was also assessed by comparing the squared root of AVE with inter-construct correlations. As showed in Table 4, the square root of AVE for each construct is higher than its correlations with other constructs, indicating acceptable discriminant validity [78].

## 5. Results

### 5.1. Results Pertaining to Question 1

After examining the measurement validity and reliability, we tested the structural equation modelling using MPLUS 8.0. The fit indices indicate a satisfactory fit for the model: Chi-square ($\chi^{2}$) is 364.7, degrees of freedom (df) is 137, $\chi^{2}/\mathrm{df}=2.66$. Root mean square error of approximation (RMSEA) is 0.07, comparative fit index (CFI) is 0.96. Standardized Root Mean Square Residual (SRMR) is 0.044. Non-normed fit index (NNFI) is 0.94. The path coefficients of the structural model were examined to assess the hypotheses. As shown in Table 5, apart from H4 and H7, the remaining seven hypotheses are supported at the significance level of 0.05. We can conclude the following.

Consumers’ attitude towards purchasing eco-labeled products, perceived behavioral control, subjective norm, and health consciousness have a positive effect on intentional purchasing behavior. As illustrated in Table 5, for example, for H1, *β* = 0.141, *p* = 0.028).

While Environmental awareness has a positive influence on consumers’ attitude towards the behavior (H4, *β* = 0.079, *p* = 0.158), the impacts of environmental awareness towards intentional purchasing behavior are not supported (H7, *β* = 0.097, *p* = 0.145).

### 5.2. Results Pertaining to Question 2

By using the built-in command statement Model Test, we try to answer Question 2, proposed in Section 1: if there exists a statistically significant difference between the total effects of HC and EA on IPB? Namely, do consumers choose eco-labeled products for concerns about the natural environment or for their health and safety?

**Figure 3:**
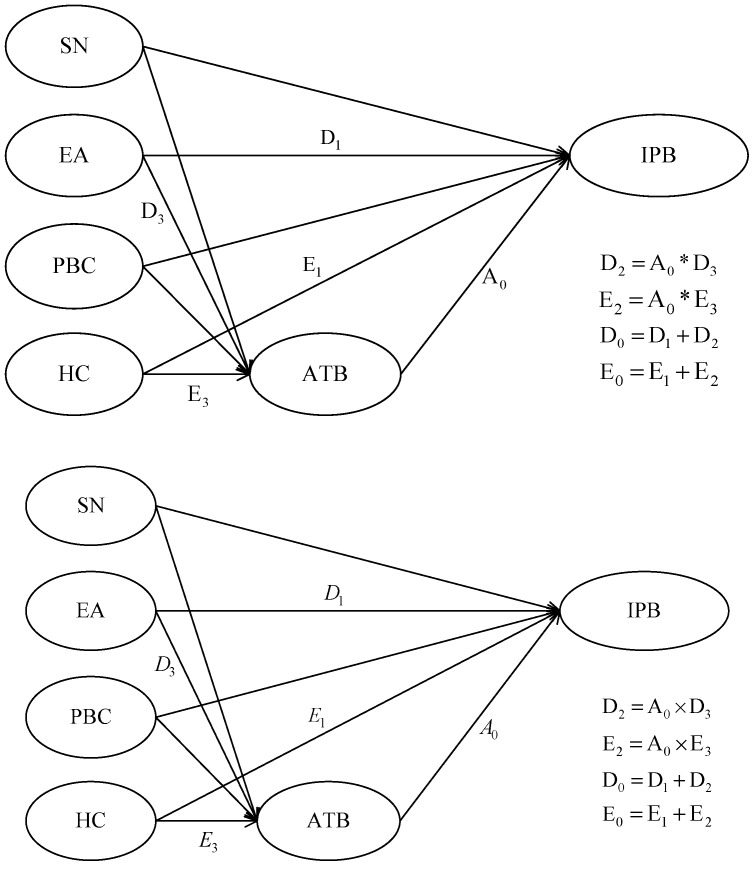
A Simplified Conceptual Framework.

As shown in Figure 3, the total effects of health consciousness (HC) on intentional purchasing behavior (IPB) include the direct effect of HC on IPB ($D_{1}$, HC→IPB) and the effect mediated by attitude towards the behavior (ATB) of consumers ($D_{2}$, HC→ATB→IPB). $D_{3}$ denotes the effect of HC on ATB (HC→ATB), $A_{0}$ denotes the effect of attitude towards the behavior on intentional purchasing behavior (ATB→IPB), $D_{2}=A_{0}\times D_{3}$. Let $D_{0}=D_{1}+D_{2}$, $D_{0}$ denote the total effects of HC on IPB.

Similarly, the total effects of environmental awareness (EA) on intentional purchasing behavior (IPB) includes the direct effect of EA on IPB ($E_{1}$, EA→IPB) and the effect mediated by attitude towards the behavior (ATB) of consumers ($E_{2}$, EA→ATB→IPB), $E_{3}$ denotes the effect of EA on ATB (EA→ATB), $E_{2}=E_{3}\times A_{0}$. Let $E_{0}=E_{1}+E_{2}$, $E_{0}$ denotes the total effects of EA on IPB. 

For contrast, $D_{0}$, $D_{1}$, $D_{2}$, $E_{0}$, $E_{1}$, $E_{2}$ were standardized before the test. According to the results of Wald test, the conclusion that $D_{0}>E_{0}$ is prominent under the confidential level of 5% (*p* = 0.038) [79].

## 6. Conclusions

### 6.1. Discussion of Findings

To understand how psychological factors influence consumers’ intentional purchasing behaviors, an extended model in line with the Theory of Planned Behavior (TPB) was built to investigate the relationships among social norm (SN), environmental awareness (EA), health consciousness (HC), perceived behavioral control (PBC), attitude towards the behavior (ATB), and intentional purchasing behavior (IPB). In addition, we also studied if consumers pay more attention to improving their health or protecting the environment when they make purchasing choices of eco-labeled products, namely, if health consciousness and environmental awareness have significantly different influences on consumers’ intentional purchasing behavior?

To this purpose, we conducted and analyzed a survey-based empirical study with 336 samples. By applying the Structural Equation Modelling (SEM), our results indicate that environmental awareness, health consciousness, and perceived behavioral control have a directly positive influence on consumers’ attitude towards purchasing eco-labeled products. Meanwhile, social norm, health consciousness, perceived behavioral control, and consumers’ attitude towards purchasing eco-labeled products have a directly positive influence on their intentional purchasing behavior.

The results show that different psychological factors have different impacts on consumers’ attitude towards purchasing eco-labeled products and consumers’ intentional purchasing behavior. To be specific, the effect of environmental awareness on consumers’ attitude towards purchasing eco-labeled products is positive and significant, but the effect of environmental awareness on consumers’ intentional purchasing behavior is insignificant. Our results provide proofs for the phenomenon that consumers’ environmental attitude and their intentional purchasing behavior are not always consistent. Namely, although consumers pay attention to environmental protection, they do not treat it as a determining factor when purchasing [32,69].

Moreover, we find that social norm has a positive influence on IPB, but the effect of it on ATB is not significant. It means that though the attitude of consumers is not influenced by the significant others around them, consumers’ intentional purchasing behaviors are influenced by their social relationships. In this case, more publicity should be conducted by the media, government agencies, and enterprises to raise the recognition of eco-labeled products. As an important external influencing factor, the efforts of news media would help to form the positive perception of the public towards buying eco-labeled products, and a favorable social environment can be helpful for promoting the buying behaviors of consumers.

Furthermore, we find that health consciousness has a stronger influence than EA on IPB. Namely, consumers pay more attention to improving their health than protecting the environment when they make purchasing choices. This result shows that the concerns to the food and commodity safety are widespread among consumers. Thus, some targeted publicity should be carried out by the government agencies and enterprises to attract those consumers who are more sensitive about their health.

### 6.2. Theoretical and Practical Implications

Our research contributes in several aspects as follows.

First, although many researches have shown that environmental awareness has a positive influence on consumers’ attitude towards eco-labeling products and purchasing intentions, the situation could be somewhat different in some developing countries, such as China. Due to the extensive food and commodity safety accidents occurred in recent years, health consciousness has been an important factor motivating consumers to choose eco-labeled products. Hence, it is necessary to investigate the motivations for Chinese consumers choosing eco-labeled products. By filling the research gap, our research can facilitate the development of better eco-labeling schemes. Government environmental policymakers can formulate more effective measures by knowing the dominant motive of consumers choosing eco-labeled products. At the same time, our results can also be a reference for business managers to attract consumers through environmental marketing.

Second, some scholars have criticized that TPB ignores one’s needs prior to engaging in a certain action, which can influence behaviors regardless of expressed attitudes [80,81]. In view of consumers’ concerns for their health as well as environmental issues, we integrated health consciousness and environmental awareness of consumers into the model, which provides a better framework to explain consumers’ attitude towards eco-labeled products and intentional purchasing behaviors.

Last but not least, we have verified that different psychological factors will influence differently on consumers’ attitude and their intentional purchasing behaviors. Namely, some psychological factors may have a positive influence on consumers’ attitude, but do not affect consumers’ intentional purchasing behaviors, and vice versa. To be specific, we find that environmental awareness has a positive influence on consumers’ attitude towards purchasing eco-labeled products, but no influence on intentional purchasing behaviors. Consumers’ attitude is not significantly influenced by social norm, but the significant others (e.g., parents, spouse, friends, teachers) would have a prominent effect on consumers’ intentional purchasing behaviors. Our results provide a new research approach to study the inconsistency of consumers’ environmental attitude and behavior [82].

### 6.3. Limitations and Future Research

Despite our efforts to conduct this research rigorously, there are some limitations in our study that should be noticed. First, limited influential factors are included in our study. Our research only focuses on these factors that could have positive influences on consumers’ intentional purchasing behavior. Some negatively related factors such as a high price, unavailability to buy, and lack of accessibility about certain information could produce totally different results, which should be considered for future research.

Furthermore, in this study, we only consider the mediating effects. But we should notice that consumers’ attitude and intentional purchasing behavior are also moderated by many factors like consumers’ cultural background, trust in the eco-labeling scheme, and product type. Hence, our research conclusions are just tenable in some certain situations.

Moreover, the value of this study is also limited for that the education level of respondents in the survey is obviously higher than the average Chinese education level, which is a flaw of this study. In addition, our convenience sample is relatively small, which cannot fully represent the Chinese population. In the future, we plan to carry out a more credible research by using larger samples and conduct a multiple group comparison among respondents with different education background.

## Figures and Tables

**Table 1:** Measurement items of constructs.

Construct	Measurement Item	Reference
Perceived Behavioral Control (PBC)	PBC1. There are many opportunities and ways in my life to buy eco-labeled products;	Giampietri et al. [53] Kikuchi-Uehara et al. [19]
	PBC2. If I wanted, I could easily purchase Eco-labeled products;	
	PBC3. Purchasing eco-labeled products depends entirely on me.	
Subjective Norm (SN)	SN1. Most people who are important to me would approve of purchasing eco-labeled products;	Giampietri et al. [53]
	SN2. Most people who are important to me want me to purchase eco-labeled products;	
	SN3. Most people who are important to me think I should purchase eco-labeled products instead of ordinary products.	
Attitude Towards the Behavior (ATB)	ATB1. I think eco-labeling schemes are good;	Davis [66]
	ATB 2. I like eco-labeled products;	
	ATB3. I support the development of eco-labeling scheme.	
Environmental Awareness (EA)	EA1. Humans are severely abusing the environment;	Dunlap et al. [67]
	EA2. If things continue on their present course, we will experience a major ecological catastrophe;	
	EA3. The balance of nature is very delicate and easily upset;	
	EA4. The earth is like a spaceship with very limited room and resources.	
Health Consciousness (HC)	HC1. I regard myself as a health consciousness consumer;	Squires et al. [68]
	HC2. I seek to choose products that are good for my health;	
	HC3. I believe that I am what I eat.	
Intentional Purchasing Behavior (IPB)	IPB1. I intend to purchase eco-labeled products within the near future;	Michaelidou & Hassan [69]
	IPB2. I would like to recommend eco-labeled products to my family and friends;	
	IPB3. How likely is it that you will purchase eco-labeled products within the next fortnight?	

**Table 2:** Demographics of the survey respondents (*N* = 336).

Characteristics	Categories	Frequency	Percent
Gender	Male	175	52.1%
	Female	161	47.9%
Age (in years)	15–24	17	5.1%
	25–34	122	36.3%
	35–44	87	25.9%
	45–54	62	18.5%
	55–64	39	11.6%
	65–74	6	1.8%
	>74	3	0.9%
Education	High school & lower	46	13.7%
	Bachelor	148	44.0%
	Master’s	120	35.7%
	Doctorate	22	6.5%
Income (CNY)	0–5 K	38	11.3%
	5–10 K	75	22.3%
	10 K–15 K	174	51.8%
	>15 K	49	14.6%
Total	/	336	100%

Note: 100 CNY (China Yuan) ≈ 15 USD.

**Table 3:** Statistics of construct items.

Constructs	Items	Standardized Loadings	CR	AVE
SN	SN1	0.86	0.94	0.84
	SN2	0.97		
	SN3	0.92		
PBC	PBC1	0.89	0.92	0.80
	PBC2	0.95		
	PBC3	0.83		
ATB	ATB1	0.82	0.89	0.73
	ATB2	0.92		
	ATB3	0.81		
EA	EA1	0.86	0.88	0.71
	EA2	0.93		
	EA3	0.90		
	EA4	0.72		
HC	HC1	0.88	0.91	0.76
	HC2	0.87		
	HC3	0.87		
IPB	IPB1	0.76	0.78	0.54
	IPB2	0.74		
	IPB3	0.71		

Note: CR stands for composite reliability, CR = (sum of standardized regression weights)^2^/[(sum of standardized regression weights)^2^ + sum of indicator error variances]. AVE stands for average variance extracted, AVE = sum of squared standardized regression weights/(sum of squared standardized regression weights + sum of indicator error variances).

**Table 4:** Constructs’ correlations and square roots of average variance extracted.

Constructs	SN	PBC	ATB	EA	HC	IPB
SN	**0.92**					
PBC	0.26	**0.89**				
ATB	0.20	0.47	**0.85**			
EA	0.10	0.45	0.47	**0.84**		
HC	0.16	0.49	0.55	0.52	**0.87**	
IPB	0.35	0.57	0.51	0.45	0.57	**0.73**

Note: Digital in bold are the square roots of AVEs of the constructs. Correlation coefficients among the six constructs are presented below the diagonal.

**Table 5:** Hypotheses and results.

Hypothesis	Estimate (*β*)	Standard Error	Critical Ratio	*p*	Result
H1	0.141	0.064	2.196	0.028	support
H2	0.202	0.075	2.692	0.007	support
H3	0.274	0.065	4.208	<0.001	support
H4	0.079	0.056	1.413	0.158	not supported
H5	0.196	0.053	3.707	<0.001	support
H6	0.187	0.064	2.918	0.004	support
H7	0.097	0.067	1.456	0.145	not supported
H8	0.343	0.069	5.001	<0.001	support
H9	0.278	0.069	4.032	0.001	support
